# Oxylipin Response to Acute and Chronic Exercise: A Systematic Review

**DOI:** 10.3390/metabo10060264

**Published:** 2020-06-25

**Authors:** Étore F. Signini, David C. Nieman, Claudio D. Silva, Camila A. Sakaguchi, Aparecida M. Catai

**Affiliations:** 1 Physical Therapy Department, Federal University of São Carlos, São Carlos, SP 13565-905, Brazil; claudiods@estudante.ufscar.br (C.D.S.); sakaguchicamila@estudante.ufscar.br (C.A.S.); mcatai@ufscar.br (A.M.C.); 2 North Carolina Research Campus, Appalachian State University, Kannapolis, NC 28081, USA; niemandc@appstate.edu

**Keywords:** inflammation, lipid mediators, lipidomics, metabolites, metabolomics, physical exercise

## Abstract

Oxylipins are oxidized compounds of polyunsaturated fatty acids that play important roles in the body. Recently, metabololipidomic-based studies using advanced mass spectrometry have measured the oxylipins generated during acute and chronic physical exercise and described the related physiological effects. The objective of this systematic review was to provide a panel of the primary exercise-related oxylipins and their respective functions in healthy individuals. Searches were performed in five databases (Cochrane, PubMed, Science Direct, Scopus and Web of Science) using combinations of the Medical Subject Headings (MeSH) terms: “Humans”, “Exercise”, “Physical Activity”, “Sports”, “Oxylipins”, and “Lipid Mediators”. An adapted scoring system created in a previous study from our group was used to rate the quality of the studies. Nine studies were included after examining 1749 documents. Seven studies focused on the acute effect of physical exercise while two studies determined the effects of exercise training on the oxylipin profile. Numerous oxylipins are mobilized during intensive and prolonged exercise, with most related to the inflammatory process, immune function, tissue repair, cardiovascular and renal functions, and oxidative stress.

## 1. Introduction

Oxylipins are oxidized polyunsaturated fatty acids (PUFAs) and function as lipid mediators for multiple physiological processes [1,2,3]. Polyunsaturated fatty acid oxidation following release from cell phospholipid membranes occurs from the activity of three enzyme systems including cyclooxygenases (COX), lipoxygenases (LOX), and cytochrome P450 (CYP) enzymes [1,4,5]. The primary fatty acids for oxylipin generation include arachidonic acid (AA), adrenic acid (AdA), linoleic acid (LA), α-linoleic acid (ALA), docosahexaenoic acid (DHA), eicosapentaenoic acid (EPA), and dihomo-γ-linolenic acid (DGLA) [1,5].

Plasma oxylipin levels can be altered in some disease states and are influenced by nutritional status and mental and physiological stressors [2,6,7,8,9,10]. Acute and chronic exercise have a strong effect on inflammation and immune function, and oxylipins may be involved at a regulatory level [2,10,11,12,13]. This potential linkage has generated interest in evaluating the effect of varying exercise workloads on oxylipin generation from COX, LOX, and CYP enzyme systems, and the interactive effects with different forms of nutritional support [10,11]. This interest has been fueled by advances in mass spectrometry (MS) and bioinformatics support that have allowed an ever increasing number of oxylipins to be measured [2]. Additionally, oxylipins are not stored but are generated by enzymatic systems in response to various types of stressors, providing a scaffold to effectively evaluate the influences of stressor doses, nutrition, obesity, medications, and other factors [2,7,8,10].

The scientific area of exercise and oxylipins is emergent, but enough studies have been published to systematically tabulate the types of oxylipins generated during different exercise workloads. The aim of this systematic review was to summarize oxylipin responses to acute and chronic exercise by their enzymatic pathways and to provide insights into potential physiological effects. The conclusions derived from this review will provide an evidence-based framework for future investigations.

## 2. Results

**Figure 1:**
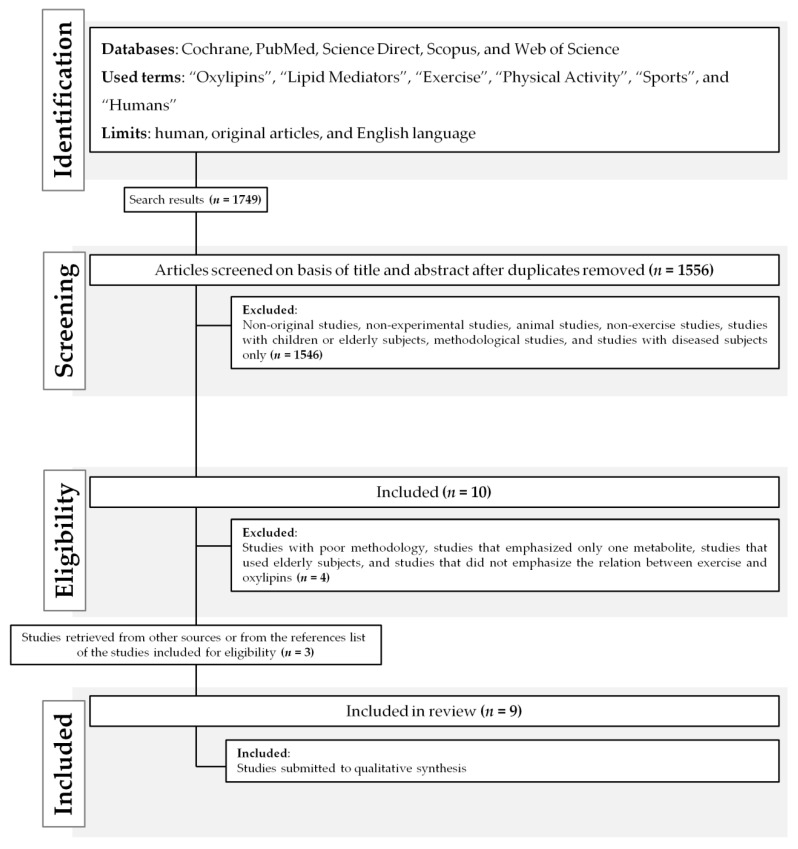
Outcomes of a review flow diagram.

After searching the literature, 1749 documents were identified (Figure 1). Nine papers were included in the final analysis after excluding duplicates and studies that did not meet the inclusion criteria (Table 1). The main reasons for exclusion were the type of study participants (animal-based, children or elderly individuals, and those with a pathology) and the lack of focus on exercise–oxylipin effects.

A scoring system was used to rank the studies for quality of research design, analysis methods, statistical support, and novelty (Table 5). Two studies were classified as having excellent quality [14,15], three as good [16,17,18], one as fair [19], and three as poor [20,21,22]. The detailed score of each study is shown in Table 1.

Table 2 summarizes the main findings and the study design of the nine selected articles. Seven studies focused on oxylipin responses to acute exercise [14,15,16,17,19,20,22] and two studies on chronic physical training [18,21]. Two studies used acute resistance exercise [16,17], one study used a graded, maximal treadmill test [19], and six studies used varying levels of acute or chronic cardiorespiratory exercise [14,15,18,20,21,22].

**Figure 2:**
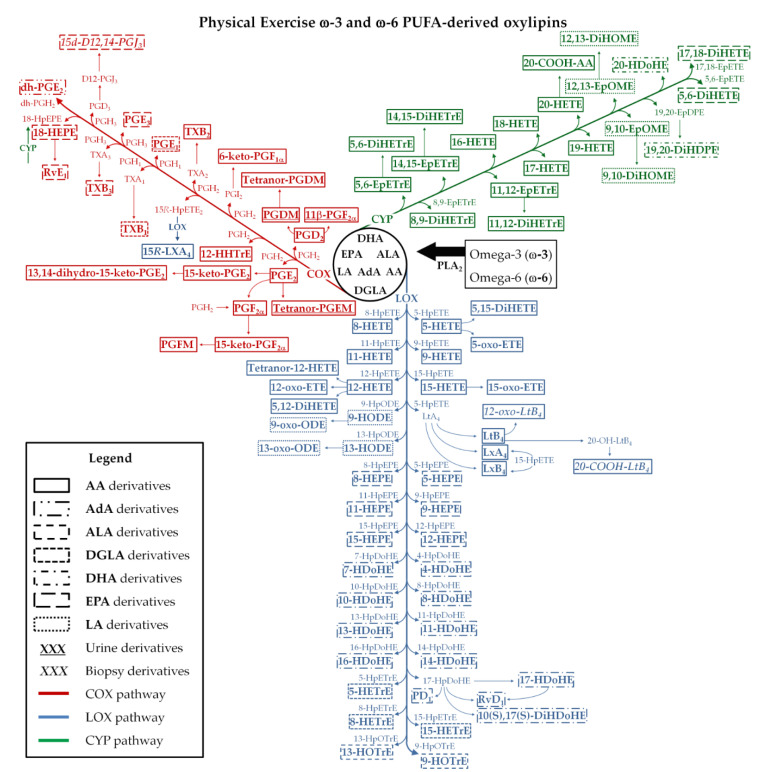
Physical exercise-related lipid pathways and metabolites. Metabolites inside the boxes are those discussed in the articles, while those outside are not discussed in the articles but are involved in the pathways. Underlined metabolites are those from urine samples. Italicized metabolites are those from muscle biopsy samples. PUFAs: polyunsaturated fatty acids; AA: aracdonic acid; AdA: adrenic acid; ALA: α-linolenic acid; 20-COOH-AA: 20-carboxy arachidonic acid; 20-COOH-LTB_4_: 20-carboxy-leukotriene B_4_; COX: cyclooxygenase; CYP: cytochrome P450; D12-PGJ_3_: Delta-12-prostaglandin J_3_; 15d-D12,14-PGJ_3_: 15-deoxy-Delta-12,14-prostaglandin J_3_; DGLA: dihomo-γ-linolenic acid; dh-PG: dihomo-prostaglandin; DHA: docosahexaenoic acid; 13,14-dihydro-15-keto-PGE_2_: 13,14-dihydro-15-keto prostaglandin E_2_; 11-dihydro-TXB_2_: 11-dihydro thromboxane B_2_; 2,3-dinoβ-11-PGF_2α_: 2,3-dinor-11β-prostaglandin F_2α_; DiHDoHE: dihydroxy-docosahexaenoic acid; DiHDPE: dihydroxy-docosapentaenoic acid; DiHETE: dihydroxy-eicosatetraenoic acid; DiHETrE: dihydroxy-eicosatrienoic acid; DiHOME: dihydroxy-octadecenoic acid; EPA: eicosapentaenoic acid; EpDPE: epoxy-docosapentaenoic acid; EpETE: epoxy-eicosatetraenoic acid; EpETrE: epoxy-eicosatrienoic acid; EpOME: epoxy-octadecenoic acid; HDoHE: hydroxy-docosahexaenoic acid; HEPE: hydroxy-eicosapentaenoic acid; HETE: hydroxy-eicosatetraenoic acid; HETrE: hydroxy-eicosatrienoic acid; HHTrE: hydroxyheptadecatrienoic acid; HODE: hydroxy-octadecadienoic acid; HOTrE: hydroxy-octadecatrienoic acid; HpDoHE: hydroperoxy-docosahexaenoic acid; HpEPE: hydroperoxy-eicosapentaenoic acid; HpETE: hydroperoxy-eicosatetraenoic acid; HpETrE: hydroperoxy-eicosatrienoic acid; HpODE: hydroperoxy-octadecadienoic acid; HpOTrE: hydroperoxy-octadecatrienoic acid; Iso: isoprostane; 15-keto-PGE_2_: 15-keto prostaglandin E_2_; 6-keto-PGF_1α_: 6-keto prostaglandin F_1α_; 15-keto-PGF_2α_: 15-keto prostaglandin F_2α_; LA: linoleic acid; LOX: lipoxygenase; LT: leukotriene; LX: lipoxin; oxo-ETE: oxo-eicosatetraenoic acid; 12-oxo-LTB_4_: 12-oxo leukotriene B_4_; oxo-ODE: oxo-octadecadienoic acid; PD_1_: protectin D_1_; PG: prostaglandin; PGDM: prostaglandin D metabolite; 11-β-PGF_2α_: 11β-prostaglandin F_2α_; PGFM: prostaglandin F metabolite; PLA_2_: phospolipase A_2_; 15*R*-HpETE: 15*R*-hydroperoxy-eicosatetraenoic acid; 15*R*-LXA_4_: 15*R*-lipoxin A_4_; Rv: resolvina; Tetranor-PGDM: tetranor-prostaglandin D metabolite; tetranor-PGEM: tetranor-prostaglandin E metabolite; TX: thromboxane.

The main metabolic pathways, the oxylipins, and the magnitude of changes reported in the studies are shown in Table 3 and Figure 2. Oxylipins, from the COX, LOX, CYP, and non-enzymatic pathways were reported from urine, serum, plasma, or muscle biopsy samples depending on the research design and MS platform. Some studies included a nutritional [14,15,18] or drug (ibuprofen) [16] intervention, and this systematic review focused on the exercises’ effects on oxylipins and not the nutrition- or ibuprofen-related findings.

García-Flores et al. [18] reported small decreases in urine oxylipin levels in 16 elite triathletes after 15 days of intense training including F_2_-isoprostanes (F_2_-IsoPs) and prostaglandin F_1α_ (PGF_1α_), and small increase in prostaglandins (PGs) 11-β-PGF_2α_, PGDM, and PGE_1_. Medina et al. [21] collected urine samples in 15 triathletes before and after a two-week period of intense training, and reported small decreases in F_2_-IsoPs and PGs (tetranor-PGEM and 11-β-PGF_2α_), and an increase in 6-keto prostaglandin F_1α_ (6-keto-PGF_1α_).

Nieman et al. [22] described large-fold increases in LA-directed hydroxyoctadecadienoic acids (9-HODE and 13-HODE), and dihydroxyoctadecenoic acids (9,10-DiHOME and 12,13-DiHOME) in 19 male cyclists after a 75 km cycling protocol. The same author in two other more recent studies, using the same 75 km cycling protocol, reported large-fold increases in plasma levels of 43 of 45 [14] and 64 of 67 [15] oxylipins. Most of the oxylipins were from AA, EPA, and DHA fatty acid substrates, with oxidation through the COX, LOX, and CYP pathways.

Giordano et al. [20] reported small increases in dihydroxyieicosatrienoic acids (8,9-DiHETrE, 11,12-DiHETrE, 14,15-DiHETrE) after 20 min of cycling exercise at 80% of the maximum load. Small increases were shown for epoxyieicosatrienoic acid (14,15-EpETrE) and 14,15-DiHETrE following 40 min of cycling at 60% of the maximum load. Gollasch et al. [19] reported small increases in dihydroxyeicosatetraenoic acids (5,6-DiHETE, 17,18-DiHETE), epoxyoctadecenoic acid (12,13-EpOME) and 5,6-DiHETrE after a maximal graded treadmill test.

Markworth et al. [16] had 16 men engage in an intensive leg resistance exercise routine, and showed small to modest increases in thromboxane B2 (TXB_2_), PGs (PGE_2_, PGD_2_, and PGI_2_), and their derivatives, leukotriene B4 (LTB_4_), resolvins (RvE_1_ and RvD_1_), isomer of protectin D1 (10(S),17(S)-DiHDoHE), 5,12-DiHETE, hydroxyeicosatetraenoic acids (12-HETE, tetranor-12-HETE, 15-HETE), 15-oxo-eicosatetraenoic acid (15-oxo-ETE), 13-oxo-hydroxyoctadecadienoic acid (13-oxo-ODE), lipoxins (LXA_4_ and LXB_4_), 11,12-DiHETrE, and 14,15-DiHETrE.

Vella et al. [17] had 12 men engage in intense knee extension resistance exercise, with muscle biopsies collected pre- and post-exercise, and then after 2 h, 4 h, and 24 h recovery. Small to moderate increases were measured for TXB_2_, PGE_2_, PGF_2α_, 15-Deoxy-Delta12,14-prostaglandin J3 (15d-D12,14-PGJ_3_), 12-oxo-leukotrieneB_4_ (12-oxo-LTB_4_), 20-carboxy leukotriene B_4_ (20-COOH-LTB_4_), 5-HETE, 12-HETE, tetranor-12-HETE, 15-HETE, 12-hydroxyeicosapentaenoic acid (12-HEPE), hydroxydocosahexanoic acids (4-HDoHE, 7-HDoHE, and 14-HDoHE) and 5,6-EpETrE, 11,12-DiHETrE and 14,15-DiHETrE.

## 3. Discussion

This systematic review provided an overview of the oxylipins that are altered with chronic exercise training or that increase after acute resistance and cardiorespiratory exercise in healthy individuals. Acute exercise induces changes in a high number of oxylipins, especially after prolonged and intensive exercise, and are generated by COX, LOX, CYP and non-enzymatic pathways from multiple fatty acid substrates (Figure 2). The specific roles of oxylipins during and after stressful levels of exercise are still being investigated, and may include regulation of inflammatory and immune system processes, vascular function, and kidney function [1,2,3,6,23,24,25,26]. This systematic review showed that the number of oxylipins generated and the fold increase is dependent on the exercise mode and workload. Plasma levels of oxylipins, even after prolonged and intensive exercise, are close to pre-exercise levels within 5 h of recovery.

### 3.1. Exercise-Related Oxylipin Formation

The release of PUFAs from cell membranes is stimulated by a group of enzymes identified as phospholipase A_2_ (PLA_2_) [1]. The PLA_2_ enzymes hydrolyze the phospholipids into fatty acids and lysophospholipids. This process may be activated when the cell is stimulated by several types of signaling pathways including mitogen-activated protein kinases (MAPKs) and extracellular signal-regulated kinases (ERK), transcriptional activators (e.g., nuclear factor-kappa B), pro-inflammatory cytokines, and other inflammatory stimuli [14,27,28,29,30,31]. These signaling pathways can be activated by exercise-induced muscle cell membrane injury and metabolic processes [32,33,34] increasing the release of PUFAs, oxylipin generation, and the inflammatory response.

The free PUFAs are subsequently oxidized by COX, LOX, and CYP enzyme pathways that generate the oxylipins [1,5]. The magnitude of increase and the diversity of oxylipins generated by these pathways from omega-6 (ω-6) and omega-3 (ω-3) free PUFAs (AA, AdA, ALA, DGLA, DHA, EPA, and LA) appear to be greatest with prolonged and intensive aerobic exercise workloads (Table 3 and Figure 2). There is scant evidence regarding the physiological roles of oxylipins within an exercise context, but the literature, in general, suggests regulatory roles in inflammatory processes, immune responses, cardiovascular system and kidney function, tissue repair, mitochondrial function, and oxidative stress [1,2,3,10,23,24,25,35,36].

The role of prostaglandins in muscle physiology, inflammation, and injury has been explored for decades [25,37,38,39,40]. The recent emergence of metabololipidomics procedures and bioinformatics support has identified a large number of oxylipins that are generated during exercise, opening up endless pathways for future research [2]. Inflammation regulation during recovery from demanding exercise bouts may emerge as a central role for many of these oxylipins [10,12]. Studies included in this review indicate that pro-inflammatory oxylipins generated during exercise include thromboxanes (TXs), PGs, HETEs, HODEs, and their derivatives (such as oxo-ETEs and oxo-ODEs), leukotrienes (LTs), DiHETrEs, and DiHOMEs (Table 3). These oxylipins are derived from AA, AdA, DGLA, and LA oxidation (Figure 2). The relationship of TXs, PGs, and LTs (such as TXB_2_, PGE_2_, and LTB_4_) with exercise has been extensively studied and are related to pro-inflammatory actions such as the increase of platelet aggregation, leukocyte activation and chemotaxis, pro-inflammatory cytokine production, vessel permeability, nociception, and changes in the vascular tone [2,25,37,38,39,41,42,43]. The recent discovery of transient but large post-exercise elevations in plasma HETEs, HODEs, DiHETrEs, and DiHOMEs underscores the complexity of this area of scientific endeavor [14,15,16,19,20,22]. These oxylipins have similar roles to TXs, PGs, and LTs, but they may also influence mitochondria respiration, skeletal muscle fatty acid uptake, myocardial and skeletal muscle blood flow, blood pressure responses, renal vessel tone and sodium excretion, and oxidative stress [2,3,19,20,22,23,24,36,44,45].

Oxylipins have physiological roles that may vary depending on the metabolic context. For example, some oxylipins are elevated with obesity and various diseases states, but may function as signaling agents during exercise [2,6,10,12]. Certain types of oxylipins exert anti-inflammatory influences to counterbalance the action of pro-inflammatory oxylipins (Table 3) [2]. These oxylipins are typically generated by ω-3 PUFAs such as ALA, DHA, and EPA, with some produced from ω-6 PUFAs [1,2] (Figure 2). Oxylipins from ω-3 PUFAs include hydroxy-octadecatrienoic acids (HOTrEs), HDoHEs, HEPEs and specialized pro-resolving mediators (SPMs), and counter pro-inflammatory actions from innate immune system cells [1,26,46]. Other physiological roles of ω-3 PUFA oxylipins include regulation of vascular tone, blood pressure, production of anti-inflammatory cytokines, tissue repair, and blood clotting [3,26,35,46,47,48]. Omega-6 PUFA oxylipins, such as 15-HETE, EpETrEs and AA lipoxins (LXs), act synergistically with ω-3 PUFA oxylipins [1,2,3,35].

Pro-resolving mediators are involved with inflammation resolution and have produced high scientific interest. They are classified into four families (i.e., lipoxins, maresins, protectins, and resolvins) [49], derived primarily from AA (15-hydroperoxy-eicosatetraenoic acid (15-HpETE)), EPA (18-HEPE), DHA (HDoHEs and hydroperoxy-docosahexaenoic acids (17-HpDoHE and 14-HpDoHE)), and play key roles in resolving inflammation in part by regulating polymorphonuclear (PMNs) leukocytes (e.g., mitigating PMN recruitment and chemotaxis), macrophages (clearance of debris and apoptotic PMNs), tissue regeneration, and nociceptive responses [10,12,26,35,50,51]. Some studies suggest that plasma levels of SPMs (e.g., LXA_4_, LXB_4_, protectins (PD_1_ and 10(S),17(S)-DiHDoHE) and resolvins (RvE_1_ and RvD_1_)) increase late in recovery from muscle-damaging exercise, and may regulate tissue regeneration and adaptation (Table 3 and Figure 2) [2,10]. Maresins may not accumulate in plasma even after demanding exercise bouts, but the large increase in 14-HDoHE suggests some involvement since both have the same precursor (14-HpDoHE) (Table 3).

Although this area of scientific endeavor is emerging, available data indicate that ω-6 and ω-3 oxylipin production is dependent on the intensity and duration of physical exercise. This observation is similar to what we have reported with plasma metabolites in general [52]. Data from the studies included in this systematic review showed small-fold changes in plasma levels of oxylipins after a single low intensity, short duration bout of exercise in contrast to large-fold changes after prolonged and intensive exercise (Table 2 and Table 3). The data also indicate that the increase of pro-inflammatory oxylipins tend to occur early in exercise recovery (TXs, PGs, HETEs and HODEs) with an increase of SPMs later in recovery [2,10]. Moreover, the studies also support that most oxylipins return to near pre-exercise levels within five hours of recovery [14,15,16,17,22]. These findings have implications for evaluating the influence of drugs and nutritional supplements on exercise-induced changes in oxylipins.

The chronic effect of physical exercise on the COX, LOX, and CYP pathways and oxylipin generation is largely unknown, and studies published thus far have numerous study design limitations [18,21]. Reported changes in plasma oxylipins with exercise training are variable and of small magnitude. Larger long-term exercise training studies are needed to confirm whether or not the typical anti-inflammatory response is supported through corresponding changes in pro- and anti-inflammatory oxylipins. Limited data indicate that obese compared to normal weight individuals have higher plasma levels of pro-inflammatory oxylipins and other related biomarkers [10,13].

### 3.2. Matrix

Six studies included in this review used blood samples (serum or plasma) for oxylipin analysis [14,15,16,19,20,22], with two using urine samples [18,21] and one using muscle biopsy samples [17]. More needs to be learned about the influence of the sample matrix on exercise-induced changes in oxylipins. The acute exercise-induced oxylipin response appeared to be comparable across serum, plasma, and muscle sample matrices. Urine samples may be more useful in long-term [53], chronic exercise training studies, with serum and plasma samples preferred for acute exercise studies due to the transient appearance of oxylipins [2,53]. The oxylipin changes following acute resistance exercise in plasma and muscle biopsy samples were somewhat comparable, but more investigation in this area is needed.

### 3.3. Limitations

Research in the area of exercise and oxylipins is relatively new, and only six studies included in this review included a large number of oxylipins measured with sensitive MS platforms. Thus, the conclusions drawn in this systematic review may change as more studies are published using a wider array of exercise modalities and workloads.

## 4. Materials and Methods

This study was conducted according to the Preferred Reporting Items for Systematic Reviews and Meta-Analyses (PRISMA) guidelines [54]. The systematized data extraction and studies selection were performed using the free standardized electronic tool State of the Art through Systematic Review (StArt) [55].

### 4.1. Search Strategy

The electronic search was performed in March 10th, 2020, using the MeSH terms selected: “Humans”, “Exercise”, “Physical Activity”, “Sports”, “Oxylipins”, “Lipid Mediators”, according to the PICOS model (Table 4) [54]. A new search was performed in May 25th, 2020, using the same terms to an update. The articles were retrieved from the databases: PubMed (via National Library of Medicine), Web of Science, SCOPUS (Elsevier), Cochrane, and Science Direct. Moreover, the search strategy was adjusted using the databases features to retrieve only human studies and English language. After the extraction from the databases, the article selection process included these steps: (1) titles and abstracts were independently examined for relevance by two researchers (EFS and CDS); (2) the full text of potentially eligible studies was reviewed. A third independent researcher (CAS) verified the inclusion process in order to solve any disagreement between the two main researchers. References of the included studies were checked for any additional relevant papers. Figure 1 represents the flow diagram of papers through the study selection process.

### 4.2. Inclusion and Exclusion Criteria

Studies were evaluated according to the listed criteria: (1) human adults and healthy population as the total or part of the sample; (2) analysis of an oxylipins list in serum, plasma, urine or muscle biopsy samples using MS; and (3) exercise as the main factor inducing changes in metabolism. Article were excluded if they focused on only one metabolite, were non-English, did not include an appropriate analysis method, included children, elderly individuals, individuals with any pathology or risk factor (such as hypertension, dyslipidemia, smoking/alcoholism and obesity), included animals only or in vitro models.

### 4.3. Data Extraction

The following data were extracted from the selected studies using procedures previously reported by our group [52]: name of the first author and year of publication, characteristics of participants and groups (i.e., population, sample size, groups, gender, age, physical activity level), research design elements (i.e., type of research, exercise mode), exercise intensity and duration, enzymatic pathway analyzed, metabololipidomics procedures (i.e., analytical platform, metabolite data), matrix, and summary comments. In addition, the oxylipins determined after physical exercise were summarized in scheme and table. Only data from the placebo or non-dietary intervention groups or phases were used.

### 4.4. Studies Quality Assessment

The quality of the studies was assessed using an adjusted scoring system created specifically for metabolomics studies [52] (see Table 5). The studies were classified according to the scores as excellent (11–9), good (8–6), fair (5–4), and poor (<4).

## 5. Conclusions

Recent improvements in mass spectrometry and bioinformatic procedures have advanced scientific understanding of oxylipins, their physiological roles, and the effects of lifestyle interventions including exercise. Exercise-induced oxylipin production during exercise has only recently been described, and the science in this area should advance greatly during the next decade. The number of oxylipins and the magnitude of increase during acute exercise bouts are directly related to the overall workload. The accumulation of oxylipins in plasma is relatively short-lived, with levels returning close to pre-exercise levels within five hours even after about three hours of intense exercise. Although physiological roles during exercise and recovery remain to be determined, data from ancillary studies suggest widespread regulatory effects centered around inflammation and vascular function. The enzyme systems involved with oxylipin generation are complex with multiple regulatory controls, and future research, such as the National Institutes of Health project, ’Molecular Transducers of Physical Activity in Humans Consortium (MoTrPAC) [56], will better define responses to chronic exercise training, moderate- versus high-intensity exercise, muscle damage, sports nutrition, and drug interventions, and varying exercise modalities.

## Figures and Tables

**Table 1:** Study classifications according to the score system in Table 5.

Investigators, Year Published	Research Design		Methodology		Novelty	Final Score	Classification
	Subjects Number	Studies Characteristics	Analysis Methods	Statistical Support			
Nieman et al. (2019) [14]	2	2	3	2	2	11	Excellent
Nieman et al. (2020) [15]	0	2	3	2	2	9	Excellent
Garcia-Flores et al. (2018) [18]	2	2	3	0	1	8	Good
Markworth et al. (2013) [16]	0	2	3	1	2	8	Good
Vella et al. (2019) [17]	0	1	3	0	2	6	Good
Gollach et al. (2019) [19]	0	1	3	0	1	5	Fair
Nieman et al.(2014) [22]	0	1	1	0	1	3	Poor
Giordano et al. (2011) [20]	0	1	1	0	1	3	Poor
Medina et al. (2012) [21]	0	0	1	0	1	2	Poor

**Table 2:** Study characteristics.

Investigators, Year Published	Study Population	Research Design	Exercise Intensity and Duration	Enzymatic Pathway	Analytical Platform	Matrix	Key Findings, Exercise Effect
Garcia-Flores et al. (2018) [18]	16 triathletes (10 men 19.0 ± 1.7 years, and 6 women 21.1 ± 3.0 years of age)	Randomized, double-blinded, placebo-controlled, and crossover design. Triathlon training at different conditions: control baseline (15 days), control training (15 days), placebo, and supplement drink crossover (100 days) and washout between these conditions (10 days), and control post-training (15 days). Training was based on objective load scale (ECOs). Urine samples timepoints: pre- and post-each condition (24 h).	Training, high-intensity, long-duration	COX, LOX, and non- enzymatic pathways	UHPLC-MS/MS	Urine	37 oxylipins detected, with small decreases in F_2_-IsoPs and PGF_1α_, and small increases in PGDM, 11-β-PGF_2α_ and PGE_1_
Medina et al. (2012) [21]	15 triathletes (10 men 19.0 ± 1.7 years and 5 women 21.8 ± 3.0 years of age)	Intense triathlon training for two weeks (cycling, swimming, running). No control group. Urine samples timepoints: pre-training (24 h) and post-training (24 h).	Training, high-intensity, long-duration	COX and non- enzymatic pathways	UPLC–QqQ-MS/MS	Urine	13 oxylipins detected, with small decreases in F_2_-IsoPs, tetranor-PGEM and 11-β-PGF_2α_, and an increase in 6-keto-PGF_1α_
Giordano et al. (2011) [20]	14 light to moderately active healthy subjects (6 men and 8 women, 36.9 ± 8.4 years of age)	In three visits, participants randomly performed submaximal bicycle tests for 20 min at 30%, 60%, and 80% of their maximal workload. In an additional visit, a test was performed at 60% of maximal work capacity for 40 min. Blood samples timepoints: pre-exercise, during exercise (20th min), and post-exercise (2 min) in the three visits, and pre-exercise and during exercise (39th min) in the additional visit	Low, moderate, and high-intensity, short-duration	CYP	UPLC-MS/MS	Plasma	6 oxylipins detected, with small increases after 80% exercise in 8,9-DiHETrE, 11,12-DiHETrE, 14,15-DiHETrE, and after 40 min 60% exercise for 14,15-EpETrE and 14,15-DiHETrE
Gollasch et al. (2019) [19]	Six healthy subjects (5 men and 1 woman 38.0 ± 15.0 years of age)	Subjects performed a maximal graded treadmill test. Blood samples timepoints: pre-exercise test (10 min before), during exercise (when heart rate reached 150 bpm), post-exercise (0 min, 10 min)	High-intensity, short-duration	LOX and CYP	HPLC-MS/MS	Plasma	56 oxylipins detected, with small increases in 5,6-DiHETrE, 12,13-EpOME, 5,6-DiHETE, 17,18-DiHETE. Most of them returned to close pre-exercise values 10 min after the end exercise
Markworth et al. (2013) [16]	16 healthy men (2 groups of 8 each, 23 ± 1.3 years and 23.0 ± 0.5 years of age)	Parallel group design randomized with two groups: placebo and ibuprofen. Exercise included a 10 min warmup; 3 sets of 8 –10 repetitions of squatting with bar; leg press 45° and knee extension at 80% 1 RM. Circuit with 1 min of recovery between sets, 3 min recovery between stations. Blood samples timepoints: after 10 h fasting; pre-exercise (15 min), post-exercise protocol (0 h, 0.5 h, 1 h, 1.5 h, 2 h, 2.5 h, 3 h, 24 h)	High-intensity resistance, short-duration	COX, LOX, and CYP	HPLC-MRM-MS/MS	Serum	87 oxylipins detected with small to moderate increases in 29 oxylipins, especially between 1 and 3 h post-exercise. Most of them returned to close pre-exercise values between 3 and 24 h after the end exercise
Nieman et al. (2020) [15]	59 healthy cyclists (38.6 ± 1.5 years of age)	Parallel group design, randomized, double blind and placebo-controlled intervention. Two-week supplementation period (freeze-dried blueberry powder or placebo) followed by a 75 km cycling time trial (while consuming water only or water with bananas). Four groups (blueberry-water trial / blueberry–banana trial / placebo-banana trial / placebo-water trial). Blood samples timepoints: pre-exercise, post-exercise (0 h, 1.5 h, 3 h, 5 h, 24 h, and 48 h)	High-intensity, long-duration	COX, LOX, and CYP	LC-MRM-MS	Plasma	Large increases in plasma concentrations of 64 of 67 oxylipins detected, with most near pre-exercise levels within 5 h post-exercise
Nieman et al. (2019) [14]	20 healthy cyclists (39.1 ± 2.4 years of age)	Randomized, crossover, counterbalanced approach. Four sessions of 75-km cycling time trial, 2-weekwashout. Four groups (Cavendish banana trial / mini-yellow banana trial / sugar beverage trial / water trial). Blood samples timepoints: pre-exercise, post-exercise (0 h, 0.75 h, 1.5 h, 3 h, 4.5 h, 21 h and 45 h)	High-intensity, long-duration	COX, LOX, and CYP	LC-MRM-MS/UHPLC/MS	Plasma	Large increases in plasma concentrations of 43 of 45 oxylipins detected, with most near pre-exercise levels within 4.5 h post-exercise
Nieman et al., (2014) [22]	19 male cyclists (38.1 ± 1.6 years of age)	Subjects performed a 75 km cycling time trial without any beverage or food containing energy or nutrients. Blood samples timepoints: pre-exercise and post-exercise (0 h, 1.5 h, 21 h)	High-intensity, long-duration	LOX and CYP	UHPLC- MS/MS and GC-MS	Plasma	Large increases in 9-HODE, 13-HODE, 9,10-DiHOME, and 12,13-DiHOME, with most near pre-exercise levels between 1.5 and 21 h post-exercise
Vella et al. (2019) [17]	12 recreationally active men (22.1 ± 0.6 years of age)	Acute bout of maximal concentric and eccentric isokinetic unilateral knee extension exercise, three sets of 12 maximal repetitions, 2 min of rest between sets. Muscle biopsy timepoints: pre-protocol, post-protocol (2 h, 4 h, 24 h)	High-intensity, resistance, short-duration	COX, LOX, and CYP	HPLC-MRM-MS/MS	Muscle Biopsy	84 oxylipins detected. Modest to small increases in 22 oxylipins at 2 h post-exercise including TXB_2_, PGE_2_, PGF_2α_, 15d-D12,14-PGJ_3_), 12-oxo-LTB_4_, 20-COOH-LTB_4_, 5-HETE, 12-HETE, tetranor 12-HETE, 15-HETE, 12-HEPE, 4HDoHE, 7-HDoHE, 14-HDoHE, 5,6-EpETrE, 11,12-DiHETrE, and 14,15-DiHETrE. Most of them returned to close pre-exercise values 4 h after the end exercise

20-COOH-LTB_4_: 20-carboxy-leukotriene B_4_; COX: cyclooxygenase; CYP: cytochrome P450; 11-dihydro-TXB_2_: 11-dehydro Thromboxane B_2_; DiHETE: dihydroxy-eicosatetraenoic acid; DiHETrE: dihydroxy-eicosatrienoic acid; DiHOME: dihydroxy-octadecenoic acid; 15d-D12,14-PGJ_3_: 15-Deoxy-D12,14-prostaglandin J_3_; EpETrE: epoxy-eicosatrienoic acid; EpOME: epoxy-octadecenoic acid; GC-MS: gas chromatography mass spectrometry; HDoHE: hydroxy-docosahexaenoic acid; HEPE: hydroxy-eicosapentaenoic acid; HETE: hydroxy-eicosatetraenoic acid; HODE: hydroxy-octadecadienoic acid; HPLC-MRM-MS/MS: high-performance liquid chromatography-multiple reaction monitoring-mass spectrometry-tandem mass spectrometer; HPLC-MS/MS: high-performance liquid chromatography-tandem mass spectrometer; IsoPs: isoprostanes; 6-keto-PGF_1α_: 6-keto Prostaglandin F_1α_; LC-MS/MS: liquid chromatography coupled to mass spectrometry; LC-MRM-MS/UHPLC: liquid chromatography-multiple reaction monitoring-mass spectrometry with ultra-high performance liquid chromatography; LOX: lipoxygenase; LT: leukotriene; 12-oxo-LTB_4_: 12-oxo Leukotriene B_4_; PG: prostaglandin; PGDM: prostaglandin D metabolite; 11-β-PGF_2α_: 11β-Prostaglandin F_2α_; tetranor-PGDM: tetranor-prostaglandin D metabolite; tetranor-PGEM: tetranor-prostaglandin E metabolite; TX: thromboxane; UHPLC-MS/MS: ultra-high performance liquid chromatography coupled to mass spectrometry; UPLC–QqQ-MS/MS: ultra-performance liquid chromatography coupled to triple quadrupole mass spectrometry; UPLC-MS/MS: ultra-performance liquid chromatography -tandem mass spectrometer.

**Table 3:** Exercise-related oxylipins measured and magnitude of changes reported in each study.

Pathway	Studies								
	Giordano et al. (2011) [20]	Medina et al. (2012) [21]	Markworth et al. (2013) [16]	Nieman et al. (2014) [22]	Garcia-Flores et al. (2018) [18]	Nieman et al. (2019) [14]	Gollach et al. (2019) [19]	Vella et al. (2019) [17]	Nieman et al. (2020) [15]
	Acute Effect (Short Duration)	Chronic Effect (Long Duration)	Acute Effect (Short Duration)	Acute Effect (Long Duration)	Chronic Effect (long Duration)	Acute Effect (Long Duration)	Acute Effect (Short Duration)	Acute Effect (Short Duration)	Acute Effect (long Duration)
COX	-	↓ Tetranor- PGEM ↓ 11-β-PGF_2α_ ↑ 6-keto-PGF_1α_	↑↑ TXB_2_ ↑↑ 12-HHTrE ↑ PGD_2_ ↑ PGE_2_ ↑ 15-keto-PGE_2_ ↑ 15-keto- PGF_2α_ ↑ 6-keto-PGF_1α_ ↑ 13,14- dihydro-15-keto-PGE_2_ ↑ RvE_1_	-	↑ PGDM ↑ 11-β-PGF_2α_ ↑ PGE_1_ ↓↓ PGF_1α_	↑↑ TXB_2_ ↑↑ 12-HHTrE ↑ PGFM ↑↑ 18-HEPE	-	↑ TXB_2_ ↑ 12-HHTrE ↑ PGE_2_ ↑ PGF_2α_ ↑ 15d- D12,14-PGJ_3_	↑↑TXB_2_ ↑↑ 12-HHTrE ↑ PGFM ↑↑ PGE_2_ ↑↑ dh-PGE_2_ ↑↑ TXB_1_ ↑↑ TXB_3_ ↑ PGE_3_ ↑ 18-HEPE ↑ RvE_1_
LOX	-	-	↑ 12-HETE ↑ 5,12- DiHETE ↑ Tetranor- 12-HETE ↑ 15-HETE ↑ 15-oxo-ETE ↑ LTB_4_ ↑ LXA_4_ ↑ LXB_4_ ↑ 9-oxo-ODE ↑ 13-HODE ↑ 13-oxo-ODE ↑ 10(S),17(S)- DiHDoHE ↑ RvD_1_	↑ 9-HODE ↑ 13-HODE		↑ 5-HETE ↑ 8-HETE ↑↑ 9-HETE ↑↑ 11-HETE ↑↑ 12-HETE ↑↑ Tetranor 12-HETE ↑↑ 15-HETE ↑↑ 9-HODE ↑↑ 9-oxo-ODE ↑↑ 13-HODE ↑ 13-oxo-ODE ↑↑ 5-HETrE ↑↑ 8-HETrE ↑↑ 15-HETrE ↑↑ 4-HdoHE ↑↑ 8-HdoHE ↑↑ 10-HdoHE ↑ 13-HdoHE ↑↑ 14-HdoHE ↑ 16-HdoHE ↑↑ 5-HEPE ↑↑ 12-HEPE ↑ 15-HEPE	-	↑ 5-HETE ↑ 12-HETE ↑↑ Tetranor 12-HETE ↑ 12-oxo- LTB_4_ ↑ 20-COOH- LTB_4_ ↑ 4-HdoHE ↑ 7-HdoHE ↑ 14-HdoHE ↑↑ 12-HEPE	↑ 5-HETE ↑ 5-oxo-ETE ↑↑ 5,15- DiHETE ↑↑ 8-HETE ↑↑ 9-HETE ↑↑ 11-HETE ↑↑ 12-HETE ↑ 12-oxo-ETE ↑↑ Tetranor 12-HETE ↑ 15-HETE ↑ 15*R*-LXA_4_ ↑↑ 9-HODE ↑↑ 9-oxo-ODE ↑↑ 13-HODE ↑↑ 13- oxo-ODE ↑↑ 5-HETrE ↑↑ 8-HETrE ↑↑ 15-HETrE ↑ 4-HdoHE ↑↑ 7-HdoHE ↑↑ 8-HdoHE ↑↑ 10-HdoHE ↑↑ 11-HDoHE
LOX	-	-				↑↑ 9-HOTrE ↑↑ 13-HOTrE	-		↑↑ 13-HDoHE ↑↑ 14-HDoHE ↑ 16-HDoHE ↑↑ 17-HDoHE ↑↑ PD_1_ ↑↑ 5-HEPE ↑ 8-HEPE ↑↑ 9-HEPE ↑ 11-HEPE ↑↑ 12-HEPE ↑↑ 15-HEPE ↑↑ 9-HOTrE ↑↑ 13-HOTrE
CYP	↑ 8,9- DiHETrE ↑ 11,12- DiHETrE ↓ 14,15- EpETrE ↑ 14,15- DiHETrE	-	↑ 11,12- DiHETrE ↑ 14,15- DiHETrE ↑ 9,10-EpOME ↑ 9,10- DiHOME	↑ 9,10- DiHOME ↑ 12,13- DiHOME	-	↑↑ 8,9- DiHETrE ↑↑ 11,12- DiHETrE ↑ 14,15- DiHETrE ↑↑ 17-HETE ↑ 18-HETE ↑ 19-HETE ↑↑ 20-HETE ↑↑ 20- COOH-AA ↑ 9,10- EpOME ↑↑ 9,10- DiHOME ↑↑ 12,13- DiHOME ↑↑ 19,20- DiHDPE ↑↑ 20- HDoHE	↑ 5,6- DiHETrE ↑ 12,13- EpOME ↑ 5,6- DiHETE ↑ 17,18- DiHETE	↑ 5,6-EpETrE ↑ 11,12- DiHETrE ↑ 14,15- DiHETrE	↑↑ 5,6- EpETrE ↑↑ 5,6- DiHETrE ↑↑ 8,9- DiHETrE ↑↑ 11,12- EpETrE ↑↑ 11,12- DiHETrE ↑↑ 14,15- DiHETrE ↑ 16-HETE ↑↑ 17-HETE ↑↑ 18-HETE ↑↑ 19-HETE ↑↑ 20-HETE ↑ 9,10- EpOME ↑ 9,10- DiHOME ↑ 12,13- DiHOME ↑↑ 19,20- DiHDPE ↑↑ 20-COOH- AA ↑↑ 20-HDoHE
Non- Enzymatic	-	↓ 8-iso- PGF_2α_	-	-	↓ 15-keto- 15-F_2t_-iso ↓ 9-epi- 15-F_2t_-iso ↓ 5-epi-5F_2t_-iso	↑↑ 5-iso- PGF_2α_-VI	-	-	↑ 8,12-iso- Isoprostane- F_2α_-VI ↑ 13,14- Dihydro-15-keto-PGF_2α_

↑↑ > 8-fold changes; ↑↑ 4 to 8-fold changes; ↑ 2 to 4-fold changes; **↑** < 2-fold changes. Late responses (after 5 h) were reported with arrow in gray scale. The arrows represent the fold changes between pre- and post-exercise condition (relative to greater difference value obtained for each metabolite). The background colors represent the metabolic pathways: COX (red), LOX (blue), CYP (green), and non-enzymatic pathway (yellow). The metabolites screened are those that had a statistical difference between the pre- and post-exercise condition. 20-COOH-AA: 20-carboxy arachidonic acid; 20-COOH-LTB_4_: 20-carboxy-leukotriene B_4_; COX: cyclooxygenase; CYP: cytochrome P450; 15d-D12,14-PGJ_3_: 15-deoxy-D12,14-prostaglandin J_3_; dh-PGE_2_: dihomo-prostaglandin E_2_; 13,14-dihydro-15-keto-PGE_2_: 13,14-dihydro-15-keto prostaglandin E_2_; 11-dihydro-TXB_2_: 11-dihydro thromboxane B_2_; 2,3-dinoβ-11-PGF_2α_: 2,3-dinor-11β-prostaglandin F_2α_; DiHDoHE: dihydroxy-docosahexaenoic acid; DiHDPE: dihydroxy-docosapentaenoic acid; DiHETE: dihydroxy-eicosatetraenoic acid; DiHETrE: dihydroxy-eicosatrienoic acid; DiHOME: dihydroxy-octadecenoic acid; EpETrE: epoxy-eicosatrienoic acid; EpOME: epoxy-octadecenoic acid; HDoHE: hydroxy-docosahexaenoic acid; HEPE: hydroxy-eicosapentaenoic acid; HETE: hydroxy-eicosatetraenoic acid; HETrE: hydroxy-eicosatrienoic acid; HHTrE: hydroxyheptadecatrienoic acid; HODE: hydroxy-octadecadienoic acid; HOTrE: hydroxy-octadecatrienoic acid; Iso: isoprostane; 15-keto-PGE_2_: 15-keto prostaglandin E_2_; 6-keto-PGF_1α_: 6-keto prostaglandin F_1α_; 15-keto-PGF_2α_: 15-keto prostaglandin F_2α_; LOX: lipoxygenase; LT: leukotriene; LX: lipoxin; oxo-ETE: oxo-eicosatetraenoic acid; 12-oxo-LTB_4_: 12-oxo leukotriene B_4_; oxo-ODE: oxo-octadecadienoic acid; PD_1_: protectin D_1_; PG: prostaglandin; PGDM: prostaglandin D metabolite; 11-β-PGF_2α_: 11β-prostaglandin F_2α_; PGFM: prostaglandin F metabolite; 15*R*-LXA_4_: 15*R*-lipoxin A_4_; Rv: resolvin; Tetranor-PGDM: tetranor-prostaglandin D metabolite; tetranor-PGEM: tetranor-prostaglandin E metabolite; TX: thromboxane.

**Table 4:** PICOS search strategy.

PICOS	
Population	Healthy adult subjects
Intervention	Physical Exercise influence
Comparator	Non-exercise/intervention condition
Outcome	Exercise-related oxylipins
Study Design	Analytical studies

**Table 5:** Score setting adjusted for lipidomics studies quality assessment.

Score Setting			
Section	Maximum Score	Aspects	Score Attribution
Research Design	2	Number of Participants	Parallel Studies 0—*n* < 20 2—*n* > 20 Crossover Studies 0—*n* < 13 2—*n* > 13
	2	Study Characteristics	—Randomized control group —Proper matrix —> 2 timepoints data collection —Duration ≥ 3wk (chronic studies only) 0—None of the previous items 1—At least 2 of the first 3 criteria listed 2—All 3 of the first 3 criteria listed
Methodology	3	Analysis Methods	1—< 10 oxylipins measured using global metabolomics 2—40–10 oxylipins measured using LC-MS/MS targeted oxylipins panel 3—> 40 oxylipins measured using LC-MS/MS targeted oxylipins panel
	2	Statistical Support	0—simple univariate statistics 1—Univariate statistics + additional analyses to sort and group the data, and to control for confounding factors 2—univariate statistics + PCA, OPLS-DA, PLS-DA, or similar advanced bioinformatics procedures
Novelty	2		0–2—New information in the literature
